# Tranexamic Acid Use in Pediatric Craniotomies at a Large Tertiary Care Pediatric Hospital: A Five Year Retrospective Study

**DOI:** 10.3390/jcm12134403

**Published:** 2023-06-30

**Authors:** Jue T. Wang, Samir C. Seshadri, Carolyn G. Butler, Steven J. Staffa, Anna S. Kordun, Karina E. Lukovits, Susan M. Goobie

**Affiliations:** Department of Anesthesiology, Critical Care, and Pain Medicine, Boston Children’s Hospital and Harvard Medical School, Boston, MA 02115, USA

**Keywords:** pediatrics, neurosurgery, patient blood management (PBM), tranexamic acid, antifibrinolytics, blood loss

## Abstract

Tranexamic acid (TXA), a synthetic antifibrinolytic drug, has proven efficacy and is recommended for major pediatric surgery to decrease perioperative blood loss. Accumulating evidence suggests that TXA reduces bleeding and transfusion in a variety of adult neurosurgical settings. However, there is a paucity of research regarding TXA indications for pediatric neurosurgery and thus, there are currently no recommendations for its use with this specific population. The objective of this study is to evaluate the existing practice of TXA administration for pediatric neurosurgery at a U.S. tertiary care pediatric hospital over a five-year period. The authors conclude that TXA administration is feasible and should be considered for pediatric neurosurgical cases where potential blood loss is a concern.

## 1. Introduction

Antifibrinolytic drugs such as tranexamic acid (TXA) and epsilon-aminocaproic acid (EACA) are synthetic lysine analogs that competitively inhibit the activation of plasminogen to plasmin, thus preventing fibrinolysis. Inhibition of fibrinolysis is imperative to minimize perioperative blood loss and avoid unnecessary allogeneic blood component transfusion. TXA is the most common antifibrinolytic used globally, has proven efficacy, and is recommended for major pediatric surgeries to decrease blood loss as part of a comprehensive perioperative blood management protocol [1]. EACA is a similar lysine analog that is useful for preventing perioperative blood loss, but due to its unavailability in numerous countries, TXA remains the most utilized. Although uncertainty regarding potential side effects, such as thromboembolic events, has limited the clinical use of TXA in the past, recently published expert consensus and clinical research offer evidence for the safety of antifibrinolytic use in adult and pediatric patients undergoing major surgery [2,3,4,5]. Additionally, accumulating evidence has suggested that TXA reduces bleeding and blood product transfusion in a variety of adult neurosurgical settings [6,7,8,9]. Intraoperative use of TXA is generally indicated for the reduction of blood loss and blood transfusion in pediatric non-cardiac and cardiac surgery. While published research focuses on the use of TXA within pediatric craniofacial, orthopedic, and cardiac surgery, there is currently no specific research pertaining to TXA use in pediatric neurosurgery, despite the potentially high risk of blood loss during neurosurgical procedures [10,11,12,13,14,15]. Antifibrinolytic use is an essential aspect of perioperative patient blood management (PBM). Physicians should feel comfortable administering TXA to pediatric patients undergoing major surgery, and dosage regimes should be based on pharmacokinetic data [16]. We present a descriptive analysis of TXA administration in pediatric patients undergoing neurosurgery at a tertiary care pediatric hospital over a five-year period. The primary aim of this retrospective cohort study is to report on the practice of TXA administration for pediatric neurosurgery at a single U.S. tertiary care pediatric hospital. A secondary aim is to present an analysis of independent associations between TXA administration, patient outcomes, and complications in pediatric neurosurgical cases.

## 2. Materials & Methods

This internal research-board-approved (IRB-P00029159) study was performed using a de-identified blood management database created using electronic medical records over a five-year period. All pediatric patients undergoing neurosurgery with a craniotomy were included. Demographic and clinical characteristics were collected using the electronic medical record, Powerchart (Cerner, London, UK). Collected data included: (1) gender, (2) age, (3) weight (kg), (4) type of craniotomy, (5) length of surgery (minutes), (6) emergent status of surgery, (7) existence of preoperative anemia (based on age-defined norms), (8) length of intensive care unit and hospital stay, (9) surgical diagnosis, and (10) adverse events or complications (per electronic medical record discharge summary ICU-10 codes). TXA administration was based on surgical and/or anesthesia provider preference. TXA dosing guidelines followed published recommendations based on pharmacokinetic modeling [17]. Exclusion criteria were non-craniotomy neurosurgical procedures and craniofacial surgery including craniosynostosis surgery.

## 3. Statistical Analysis

Retrospective data on patients who underwent neurosurgical craniotomy procedures were analyzed. Continuous data are presented as median, interquartile range (IQR), and range, and categorical data are presented as frequency (*n*) and percentage (%). Univariate testing was performed using the Wilcoxon rank sum test for continuous variables or Fisher’s exact test for categorical variables. Multivariable regression analysis was performed to determine the independent association between TXA and outcomes while adjusting for blood product transfusion, American Society of Anesthesiologists (ASA) physical status classification, and length of procedure, using median regression for continuous outcomes and logistic regression for binary outcomes. Results from the multivariable analysis are presented as adjusted coefficients or odds ratios with corresponding 95% confidence intervals and *p*-values. Statistical analyses were performed using Stata (version 16.0, StataCorp LLC, College Station, TX, USA). A two-tailed *p* < 0.05 was used for determining statistical significance.

## 4. Results

From January 2017 to October 2021, 1171 pediatric craniotomies were performed (Table 1). The majority of patients were school-age children (median age 9.3 years). The median ASA status was III (59.4%) and there was roughly an even gender distribution of 46.1% female and 53.9% male patients. The majority of patients did not have preoperative anemia (948/1171, 81%), but 10.7% had mild anemia, 7.9% had moderate anemia and 0.4% had severe anemia (based on the World Health Organization Hemoglobin Concentrations for the Diagnosis of Anemia and Assessment of Severity. Vitamin and Mineral Nutrition Information System. World Health Organization, Geneva) [18]. A range of neurosurgical craniotomy procedures were performed, with TXA given in 34.5% of all craniotomies. The sub-categories of craniotomy types and etiologies were as follows: 84.8% hemispherectomies, 68.5% seizure surgeries, 46.2% brain tumor surgeries, 34% arteriovenous malformation repair, 25.7% trauma surgeries, 25% other neurosurgical procedures, 15.4% posterior fossa surgeries, and 12.5% encephalocele repairs (Table 2). The indications for TXA were guided by provider preference and based on expert consensus recommendations [17]. TXA dosage was within pharmacokinetic modeling guidelines based on published PK data and as per our Boston Children’s Hospital TXA protocol; TXA dosage range is a loading dose over 15 min of 10–30 mg/kg (maximum 2 g) followed by an infusion of 5–10 mg/kg/h [17].

The overall allogeneic blood component transfusion rate was 15%, with 14% receiving red blood cells and 5% receiving ‘yellow’ products; fresh frozen plasma, platelets, and/or cryoprecipitate. Hemispherectomies and craniotomies performed for trauma surgery were the highest transfused category at a rate of 37% each (Table 2).

In patients with tranexamic acid given (*n* = 404), 108 patients (26.7%) had blood products transfused, 107 patients (26.5%) had red blood products transfused and 44 patients (10.9%) had yellow blood products transfused. In the non-tranexamic patients (*n* = 767), 68 patients (8.9%) had blood products transfused, 59 patients (7.7%) had red blood products transfused and 13 patients (1.7%) had yellow blood products transfused (Table 3).

The median length of hospital stay in the entire cohort was 5 days (IQR 4, 9), which was the same as those in the group that did not receive TXA (5 days, IQR 4, 7), but was longer in the group that did receive TXA: 7 days (IQR 5, 13) which is a statistically significant difference (*p* < 0.001). The median intensive care unit (ICU) length of stay was 0.9 days (IQR 0, 1.2) for the entire cohort. The median ICU length of stay for those patients who did not receive TXA was 0.87 (IQR 0, 1.14), and 0.97 (IQR 0.76, 1.92) which is also statistically significant (Table 4).

Patient outcomes and complications are listed in Table 4. Notable complications included respiratory failure, pulmonary edema, and fever. The rate of respiratory failure was higher in the TXA group compared to the non-TXA group (11.39% vs. 6.78%, *p* = 0.008). Pulmonary edema was also more prevalent in the TXA group (3.47% vs. 1.3% *p* = 0.017), as was fever (14.1% vs. 6.39% *p* < 0.001). Multivariable analysis of outcomes significant upon univariate testing (Table 5) controlling for perioperative blood transfusion, ASA status, and length of surgical procedure reports an independent association between TXA use and increased hospital length of stay. TXA was not associated with any increased complications including thromboembolic events; the overall rate of thrombosis/embolism was reported as 1.6%.

Multivariate analysis of TXA versus outcomes and complications in craniotomies (Table 5) revealed only statistical significance in the length of hospital stay (odds ratio 0.99, *p* = 0.008), but not the length of ICU stay. There was no statistical significant difference in the rate of respiratory failure, pulmonary edema, thromboembolic events, or fever.

## 5. Discussion

Major or clinically significant blood loss during pediatric neurosurgery can lead to anemia, hemodynamic instability, and decreased end-organ perfusion/oxygenation, thus necessitating blood component transfusion. Given that utilization of TXA has been shown to reduce intraoperative bleeding in various types of surgical settings, its use is recommended for all pediatric surgeries that may involve clinically significant blood loss (>20% total blood volume). TXA should be used in tandem with other patient blood management strategies, such as, but not limited to, timely diagnosis and correction of preoperative anemia with iron supplementation, restrictive transfusion strategies, intraoperative use of cell-saver, use of point-of-care-guided management of coagulopathy by viscoelastic testing and, of course, meticulous surgical hemostasis [11,17]. 

The results of this study report the routine clinical use of TXA in a variety of pediatric neurosurgical procedures (craniotomies) performed at a tertiary care pediatric hospital. This study has limitations which include the retrospective nature of the design and the fact the decision to utilize TXA was guided by provider preference (based on TXA published recommended indications and expert consensus guidelines) Therefore, TXA was likely administered to the higher risk pediatric neurosurgical patients (for example 84.8% of hemispherectomy patients received TXA; this surgical procedure has the highest rates of blood loss and transfusion with the most extensive surgery). This could potentially be one contributing factor in our finding that the length of hospital stay was longer in the TXA group compared to the non-TXA group (Table 2 and Table 4). Of note, another limitation of the report is that one of the patient populations with the highest rates of hemorrhagic complications, specifically neonates/infants, represented a very small percentage of this neurosurgical cohort. Finally, it must be noted that 7.9% of patients included in this cohort were in the adult age category, a median age of 20.2 years, and a median weight of 72.6 kg (Table 1) as they continued their care with pediatric specialists at this pediatric institution.

One historical limiting factor for the routine use of TXA was the uncertainty of the risk-benefit ratio weighing the risk of bleeding versus the venous thromboembolism (VTE) risk [19,20]. Large multicenter trials in adults in various settings have consistently reported no increased thromboembolic risk [21,22,23,24]. VTE risk is perhaps a unique concern for those undergoing surgery for brain tumor resection given the already theoretical increased risk of VTE due to hypercoagulability secondary to malignancy [25]. However, there is mounting high-quality evidence that, in patients at higher risk for VTE including those with malignancy, there is no increased risk of thrombosis [25]. In a systematic review and meta-analysis, the use of TXA was associated with reduced all-cause mortality without increased risk of venous or arterial thrombotic complications in adult cancer and high-risk patients [26]. In a study of adult patients surgically treated for vertebral column tumors, Pennington et al. found that TXA was not associated with increased VTE risk, although high-dose TXA (≥20 mg/kg) was associated with increased odds of deep vein thrombosis (DVT) or pulmonary embolism (PE) [25]. A recent systematic review of antifibrinolytic use in adult patients (*n* = 408 patients in 7 studies) undergoing spinal surgery for oncological spinal disease reports efficacy with no increased risk of DVT or PE [27]. While this information cannot be directly extrapolated and applied to neurosurgical pediatric populations, our small single center study supports this in reporting no increased thromboembolic events related to TXA in this cohort.

Fifteen percent of all craniotomies in our report received allogeneic blood components perioperatively, with the highest rates of transfusion in hemispherectomies and trauma surgery. The effect of TXA on reducing blood transfusion in our cohort cannot be concluded as this is a descriptive report. However, efficacy has been shown in other pediatric non-cardiac surgery such as cranial vault remodeling surgery and major orthopedic surgery [5,10,13,28]. Furthermore, Spinella et al. and the MATIC investigators (MAssive Transfusion epidemiology and outcomes In Children) report that the administration of antifibrinolytic medications during life-threatening events was independently associated with improved 6- and 24-h survival in bleeding children [29]. The authors recommend that consideration should be given to the use of antifibrinolytics in pediatric patients with life-threatening hemorrhage. The ongoing Traumatic Injury Clinical Trial Evaluating Tranexamic Acid in Children (TIC-TOC) will report the effects of TXA in children with severe trauma and hemorrhagic injuries (ClinicalTrials.gov registration number: NCT02840097).

This retrospective descriptive cohort study demonstrates the practice and the feasibility of TXA administration, dosed within pharmacokinetic modeling parameters, to reduce blood loss in high-risk children undergoing craniotomies. As the decision to administer TXA was based on provider preference within published guidelines, higher-risk neurosurgical patients likely received TXA, therefore no conclusions can be drawn regarding the association of TXA with patient-centered outcomes in this report. Further prospective multicenter research should address the efficacy and safety of its use in this setting of pediatric neurosurgery.

## Figures and Tables

**Table 1:** Patient demographics of craniotomies in the entire cohort and by tranexamic acid (TXA) administration. Continuous data are presented as median (interquartile range) and range. Categorical data are presented as *n* (%). Abbreviations: TXA, tranexamic acid; ASA, American Society of Anesthesiologists Physical Status Classification.

Variable	Entire Cohort	TXA Given	TXA Not Given
Number of Cases	1171	404	767
Age (years)	9.3 (4.2, 14.4) [0.01, 31.4]	7.9 (2.6, 13.3) [0.01, 29.4]	9.9 (5, 14.8) [0.01, 31.4]
Age Category			
Neonatal	11 (0.9%)	4 (1%)	7 (0.9%)
Infant	62 (5.3%)	34 (8.4%)	28 (3.7%)
Preschool	278 (23.7%)	120 (29.7%)	158 (20.6%)
School-aged	384 (32.8%)	116 (28.7%)	268 (34.9%)
Adolescent Female	169 (14.4%)	57 (14.1%)	112 (14.6%)
Adolescent Male	174 (14.9%)	50 (12.4%)	124 (16.2%)
Adult	93 (7.9%)	23 (5.7%)	70 (9.1%)
Weight (kg)	32.7 (16.8, 55.9) [2.3, 172]	28.2 (14.1, 49.6) [2.3, 172]	35 (19.3, 59.2) [2.9, 138]
ASA *			
I	21 (1.8%)	4 (1%)	17 (2.2%)
II	297 (25.4%)	61 (15.1%)	236 (30.8%)
III	695 (59.4%)	269 (66.6%)	426 (55.5%)
IV	144 (12.3%)	62 (15.4%)	82 (10.7%)
V	14 (1.2%)	8 (2%)	6 (0.8%)
Gender			
Female	540 (46.1%)	172 (42.6%)	368 (48%)
Male	631 (53.9%)	232 (57.4%)	399 (52%)
Type of Procedure			
Brain Tumor Resection	371 (31.7%)	171 (42.3%)	200 (26.1%)
Moya Moya (Pial Synangiosis)	151 (12.9%)	0 (0%)	151 (19.7%)
Seizure **	127 (10.9%)	87 (21.5%)	40 (5.2%)
Hemispherectomy (anatomical or functional)	59 (5%)	50 (12.4%)	9 (1.2%)
Aneurysm	47 (4%)	16 (4%)	31 (4%)
Encephalocele	16 (1.4%)	2 (0.5%)	14 (1.8%)
Posterior Fossa	234 (20%)	36 (8.9%)	198 (25.8%)
Trauma	70 (6%)	18 (4.5%)	52 (6.8%)
Other	96 (8.2%)	24 (5.9%)	72 (9.4%)
Length of Surgery (minutes)	247 (157, 415) [22, 1951]	406 (284, 512) [70, 1106]	198 (136, 308) [22, 1951]
Emergent	254 (21.7%)	108 (26.7%)	146 (19%)
Preoperative Anemia ***			
Not Anemic	948 (81%)	313 (77.5%)	635 (82.8%)
Mild Anemia	125 (10.7%)	53 (13.1%)	72 (9.4%)
Moderate Anemia	93 (7.9%)	34 (8.4%)	59 (7.7%)
Severe Anemia	5 (0.4%)	4 (1%)	1 (0.1%)

Continuous data are presented as median (interquartile range) [minimum, maximum], and categorical data are presented as *n* (%). * ASA = American Society of Anesthesiologists Physical Status Classification. ** Seizure surgical procedure = Focal resection, subdural grids and strips placement and/or removal & resection seizure focus. *** Preoperative Anemia = Anemia based on WHO 2011; http://www.who.int/vmnis/indicators/haemoglobin.pdf, accessed on 18 November 2021.

**Table 2:** Patient blood management variables for types of craniotomies in the entire cohort. Data are presented as number (*n*) and percent (%) of the 1171 total general anesthesia encounters performed for craniotomies.

Type of Surgical Procedure	Tranexamic Acid Administered	Total Blood Products Transfused	Yellow Blood Products Transfused *	Red Blood Products Transfused **
All Procedures (*n* = 1171)	404 (34.5%)	176 (15%)	57 (4.9%)	166 (14.2%)
Brain Tumor (*n* = 371)	171 (46.1%)	50 (13.5%)	17 (4.6%)	48 (12.9%)
Moya Moya (*n* = 151)	0 (0%)	7 (4.6%)	0 (0%)	6 (4%)
Seizure (*n* = 127)	87 (68.5%)	22 (17.3%)	6 (4.7%)	21 (16.5%)
Hemispherectomy (*n* = 59)	50 (84.8%)	22 (37.3%)	9 (15.3%)	20 (33.9%)
Aneurysm (*n* = 47)	16 (34%)	4 (8.5%)	0 (0%)	6 (12.8%)
Encephalocele (*n* = 16)	2 (12.5%)	3 (18.8%)	1 (6.3%)	3 (18.8%)
Posterior Fossa (*n* = 234)	36 (15.4%)	22 (9.4%)	8 (3.4%)	23 (9.8%)
Trauma (*n* = 70)	18 (25.7%)	26 (37.1%)	11 (15.7%)	25 (35.7%)
Other (*n* = 96)	24 (25%)	20 (20.8%)	5 (5.2%)	14 (14.6%)

Data are presented as *n* (row %). * Yellow Blood Products = fresh frozen plasma, platelets, and/or cryoprecipitate. ** Red Blood Products = packed red blood cells.

**Table 3:** Patient blood management variables for craniotomies in the entire cohort and by tranexamic acid (TXA) administration. Data are presented as number (*n*) and percent (%) of the 1171 total general anesthesia encounters performed for craniotomies. Abbreviations: TXA, tranexamic acid.

Outcome	Entire Cohort (*n* = 1171)	TXA Given (*n* = 404)	TXA Not Given (*n* = 767)	*p* Value
Total Blood Products Transfused	176 (15%)	108 (26.7%)	68 (8.9%)	<0.001 *
Yellow Blood Products Transfused **	57 (4.9%)	44 (10.9%)	13 (1.7%)	<0.001 *
Red Blood Products Transfused ***	166 (14.2%)	107 (26.5%)	59 (7.7%)	<0.001 *

Data are presented as *n* (%). *p* values were calculated using Fisher’s exact test. * Statistically significant. ** Yellow Blood Products = fresh frozen plasma, platelets and/or cryoprecipitate, *** Red Blood Products = packed red blood cells.

**Table 4:** Univariate comparison of outcomes and complications for craniotomies by tranexamic acid (TXA) administration. Data are presented as number (*n*) and percent (%) of the 1171 total general anesthesia encounters performed for craniotomies with tranexamic acid administration for 404 encounters. Hospital and intensive care unit length of stay is measured in days. Complications are taken from the electronic medical record. Complications reported include (1) allergic reaction, (2) cardiac arrest, (3) heart failure, (4) neonatal cardiac failure, (5) pulmonary infection, (6) thrombosis or embolism, (7) respiratory failure, (8) respiratory arrest, (9) acute respiratory distress syndrome, (10) pulmonary edema, (11) fever, (12) renal failure, (13) sepsis, (14) transfusion-associated anaphylactic reaction, (15) other complication not specified. Abbreviations: TXA, tranexamic acid; ICU, intensive care unit; ARDS, acute respiratory distress syndrome.

Outcome	Full Cohort (*n* = 1171)	TXA Given (*n* = 404)	TXA Not Given (*n* = 767)	*p* Value
Length of hospital stay (days)	5 (4, 9) [0, 161]	7 (5, 13) [0, 115]	5 (4, 7) [0, 161]	<0.001 *
Length of stay in ICU (days)	0.9 (0, 1.2) [0, 87.1]	0.97 (0.76, 1.92) [0, 87.1]	0.87 (0, 1.14) [0, 41.8]	<0.001 *
Complications (any)	187 (16%)	90 (22.3%)	97 (12.7%)	<0.001 *
Allergic Reaction	1 (0.09%)	1 (0.25%)	0 (0%)	0.345
Cardiac Arrest	6 (0.51%)	4 (0.99%)	2 (0.26%)	0.19
Heart Failure	8 (0.68%)	3 (0.74%)	5 (0.65%)	0.999
Neonatal Cardiac Failure	1 (0.09%)	1 (0.25%)	0 (0%)	0.345
Pulmonary Infection	30 (2.56%)	10 (2.48%)	20 (2.61%)	0.999
Thrombosis / Embolism	19 (1.62%)	9 (2.23%)	10 (1.3%)	0.234
Respiratory Failure	98 (8.37%)	46 (11.39%)	52 (6.78%)	0.008 *
Respiratory Arrest	2 (0.17%)	0 (0%)	2 (0.26%)	0.548
ARDS (Acute Respiratory Distress Syndrome)	4 (0.34%)	3 (0.74%)	1 (0.13%)	0.121
Pulmonary Edema	24 (2.05%)	14 (3.47%)	10 (1.3%)	0.017 *
Fever	106 (9.05%)	57 (14.1%)	49 (6.39%)	<0.001 *
Renal Failure	8 (0.68%)	5 (1.24%)	3 (0.39%)	0.133
Sepsis	14 (1.2%)	4 (0.99%)	10 (1.3%)	0.782
Transfusion Associated Anaphylactic Reaction	0 (0%)	0 (0%)	0 (0%)	0.999
Other	2 (0.17%)	0 (0%)	2 (0.26%)	0.548

Continuous data are presented as median (interquartile range) [minimum, maximum] and categorical data are presented as *n* (%). *p* values were calculated using the Wilcoxon rank sum test or Fisher’s exact test, as appropriate. * Statistically significant.

**Table 5:** Multivariable analysis of tranexamic acid (TXA) administration versus outcomes and complications in craniotomies. Data are presented as an adjusted coefficient or odds ratio for tranexamic acid administration for 404 craniotomies. Hospital and intensive care unit length of stay is measured in days. Complications are taken from the electronic medical record. Complications reported include (1) thrombosis or embolism, (2) respiratory failure, (3) pulmonary edema, and (4) fever. Abbreviations: TXA, tranexamic acid; ICU, intensive care unit.

Outcome	Adjusted Coefficient or Odds Ratio for TXA	95% CI	*p* Value
Length of hospital stay (days)	0.99	(0.25, 1.72)	0.008 *
Length of stay in ICU (days)	0.07	(−0.11, 0.25)	0.472
Complications (any)	0.99	(0.66, 1.47)	0.948
Thrombosis Embolism	1.21	(0.42, 3.49)	0.731
Respiratory Failure	0.98	(0.59, 1.6)	0.924
Pulmonary Edema	1.31	(0.53, 3.26)	0.557
Fever	1.28	(0.81, 2.04)	0.288

For hospital and ICU length of stay, multivariable median regression was implemented. For complications, multivariable logistic regression was implemented. Multivariable analysis included outcomes significant on univariate testing; other outcomes were too rare (low percent rates). Each model is adjusted for blood products transfused, ASA, and length of the procedure. * Statistically significant.

## Data Availability

The data presented in this study are available on request from the corresponding author.
